# Biomolecular Condensates Decipher Molecular Codes of Cell Fate: From Biophysical Fundamentals to Therapeutic Practices

**DOI:** 10.3390/ijms25074127

**Published:** 2024-04-08

**Authors:** Xing Sun, Yangyang Zhou, Zhiyan Wang, Menglan Peng, Xianhua Wei, Yifang Xie, Chengcai Wen, Jing Liu, Mao Ye

**Affiliations:** 1 Molecular Science and Biomedicine Laboratory, State Key Laboratory of Chemo/Biosensing and Chemometrics, College of Biology, College of Chemistry and Chemical Engineering, Aptamer Engineering Center of Hunan Province, Hunan University, Changsha 410082, China; xsun@hnu.edu.cn (X.S.); yangyang.zhou@universite-paris-saclay.fr (Y.Z.); s222201397@hnu.edu.cn (Z.W.); pml@hnu.edu.cn (M.P.); xianhuaw@hnu.edu.cn (X.W.); 2 Molecular Biology Research Center and Center for Medical Genetics, School of Life Sciences, Central South University, Changsha 410000, China; 212511063@csu.edu.cn (Y.X.); kongqing@csu.edu.cn (C.W.)

**Keywords:** biomolecular condensates, physical trait, molecular behavior, cell fate, diseases

## Abstract

Cell fate is precisely modulated by complex but well-tuned molecular signaling networks, whose spatial and temporal dysregulation commonly leads to hazardous diseases. Biomolecular condensates (BCs), as a newly emerging type of biophysical assemblies, decipher the molecular codes bridging molecular behaviors, signaling axes, and clinical prognosis. Particularly, physical traits of BCs play an important role; however, a panoramic view from this perspective toward clinical practices remains lacking. In this review, we describe the most typical five physical traits of BCs, and comprehensively summarize their roles in molecular signaling axes and corresponding major determinants. Moreover, establishing the recent observed contribution of condensate physics on clinical therapeutics, we illustrate next-generation medical strategies by targeting condensate physics. Finally, the challenges and opportunities for future medical development along with the rapid scientific and technological advances are highlighted.

## 1. Introduction

A cell can be vividly perceived as a sophisticated and highly ordered molecular machine. Precisely organized molecular networks of biochemical reactions and/or biophysical interactions exert formidable effects on the physiological and pathological fate of cells including survival, metabolism, development, migration, and death. The accurate and concerted organization of the biomolecular state makes significant contributions in the complex and crowded intracellular environment, as it commonly reflects the overall molecular properties in terms of molecular mobility, dynamics, activity, specificity, and spatiotemporal availability. Growing evidence has illustrated the biological and clinical significance of biomolecular condensates (BCs), formed through a process called phase separation.

BCs are single- or multiple-component assemblies of proteins and nucleic acids, as well as other small components, such as lipids and inorganic ions. Their sizes normally range from hundreds of nanometers to several micrometers. Although the first discovery of intracellular condensates, the nucleolus, dates back to one-and-a-half centuries ago; however, the physical traits and resulting powerful biological functions were not fully illuminated until the primordial delineation of the physical nature of P granules in *Caenorhabditis elegans* by Hyman et al. [1]. An increase in academic advancements in BCs further emphasizes their physical properties and extensive contribution to cell biology, diseases and biomedicine [1,2,3,4,5], of which a series of basic cell events, such as enzymatic activity, gene expression, organelle formation, cell adhesion, migration, immune responses and drug resistance, have been exhaustively discussed [2,6,7,8,9,10,11,12].

In cells, BCs generally present as (para)speckles, granules, droplets, and some amorphous aggregations [13,14,15,16]. At the micron or submicron level, condensate assemblies are endowed with superior physical traits over smaller protein multimers and larger bulk aggregations. Firstly, opening up new intracellular compartments creates special spaces for the faithful execution of specific reactions or interactions at proper time, and condensates of these sizes exhibit both a high surface-to-volume ratio and appropriate steady internal spaces, thereby generating versatile function-specialized platforms for intermolecular crosstalk. Moreover, condensates share typical physical features of soft matter, thus adding new insights into the acting mode in molecular signaling axis. In particular, in the process of mixing or demixing, several patterns of forces are generated or diminished, which in turn exert significant effects on the molecular events. Furthermore, due to the limited number (normally ranging from hundreds to several thousands) of biomolecules involved in the finite spatiotemporal spaces, functions of BCs seem to be more flexible and specific. Overall, BCs can be considered as physical organizers of cell fate.

However, a comprehensive summary of how the molecular physics of BCs are interpreted into the modulation of cell and molecular biology is lacking. In this review, we highlight the recent updates of the physical features of BCs, focus on the functions and contributing factors of these altered biophysics, and finally discuss their underlying significance to clinical applications.

## 2. Physical Traits of BCs for Molecular Signaling

**Figure 1:**
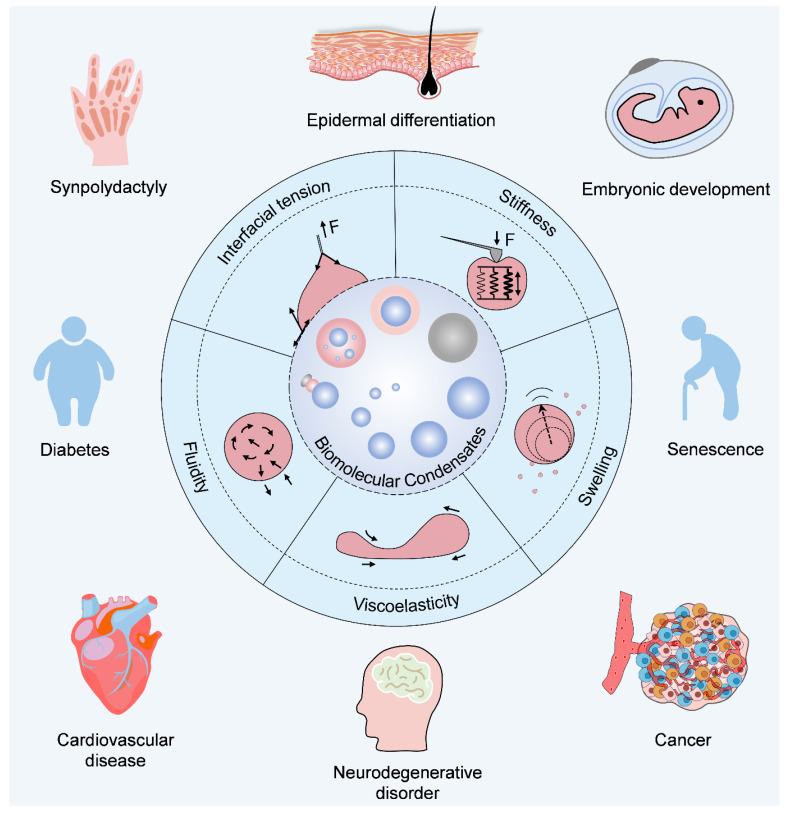
Physical traits of biomolecular condensates and their effects on development, differentiation and diseases. Because of differences in their composition and biological contexts, biomolecular condensates show versatile sizes, shapes, and interactive states, which collectively induce five representative physical traits, namely, interfacial tension, stiffness, viscoelasticity, fluidity and swelling. As suggested by the recent studies, these proposed physical traits of biomolecular condensates play a crucial role in controlling embryonic development, differentiation, and senescence, as well as the onset and progression of a broad variety of diseases such as synpolydactyly, diabetes, cancer, neurodegeneration disorders and cardiovascular diseases. Taken together, it is not only the molecular-level chemical and biological states of biomolecules, but also mesoscopic-level physical traits by forming biomolecular condensates that dictate cell fate in both physiological and pathological conditions.

**Figure 2:**
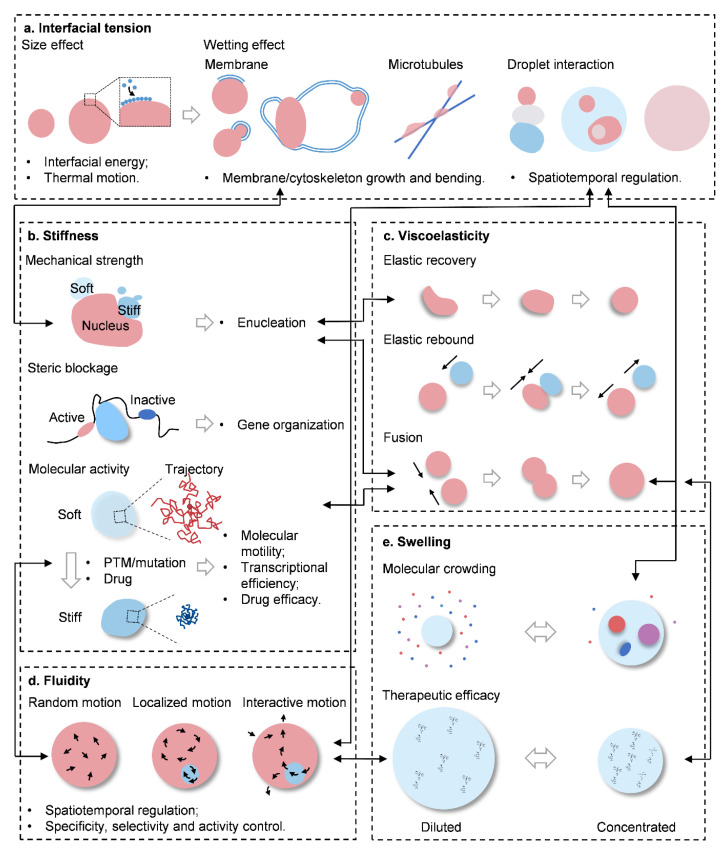
Representative working modes of physical traits of biomolecular condensates. (**a**) Interfacial tension determines the size of condensates, at which length it forms a proper platform for biological functions. Moreover, interfacial tension allows for the wetting of condensates on membranes and microtubules, functioning in their formation, elongation, curvature and stability. Wetting can also govern droplet interaction ruling the spatiotemporal allocation of biomolecules. (**b**) Stiffness is the mechanical property of condensates. With enough mechanical strength, condensates can deform adjacent structures as nucleus, and serve as a robust insulator for the steric blockage of active and inactive regions. Moreover, molecular activities in soft and stiff condensates differ significantly, leading to different outcomes in function and efficacy. (**c**) Viscoelasticity describes the micro-rheological feature of condensates. Through elastic recovery and rebound, condensates maintain constitutive stability under mechanical stress or heterogenic collisions. Upon homogenic collisions, condensates can maintain molecular concentration and functional efficiency through fusion. (**d**) Fluidity reflects molecular dynamic in condensates, which orchestrates the spatiotemporal distribution, and influences the specificity, selectivity and activity of biomolecules. (**e**) Swelling, or shrinkage, is the volume change of condensates. With this trait, condensates can buffer molecular crowding under stress and affect therapeutic efficacy. Taken together, physical traits of condensates do co-exist and can be intensely associated for functions, and bridging molecular profiles with physical traits may become another hotspot in both academic and translational research.

Due to unique constitutive coalescence, soft matter properties, size effects, and molecular dynamics, a variety of extraordinary physical traits are generated, of which interfacial tension [7,17,18,19], stiffness [2,4,20], viscoelasticity [21,22,23,24], fluidity [25,26], and swelling [27,28] represent the most characteristic features, thereby regulating physiological and pathological events (Figure 1 and Figure 2).

## 3. Interfacial Tension

The most characteristic feature of BCs is opening up a set of new compartments, with a massive number of interfaces in the crowded and a chaotic intracellular environment. Imbalanced forces of attraction for the molecules at the interfaces of two immiscible phases cumulatively give rise to the interfacial tension against the mixing phase. Increasing evidence has suggested interfacial tension to be one of the mainstay physical traits of BCs [29]. Statistically, the interfacial tension of condensates is around 0.4–10 μN/m, while those of colloid liquids are typically 0.1 μN/m and below [1,30,31]. It explains why BCs are commonly shown as spherical droplets, with a size ranging from hundred nanometers to several micrometers in diameter. From the point of mesoscopic view, intracellular immiscible condensates always tend to spontaneously minimize the overall interfacial energy to maintain thermal stability of the system, and eventually leading to several aspects of physical alternation, which can be primarily summarized into size effects and wetting (or in some scenarios called capillarity) (Figure 2a).

### 3.1. Size Effects

Size is a principal element in shaping biomolecular functions. Scaled at a micron or submicron level, BCs are generally endowed with typical size effects that introduce several aspects of benefits for cell biology.

**Figure 3:**
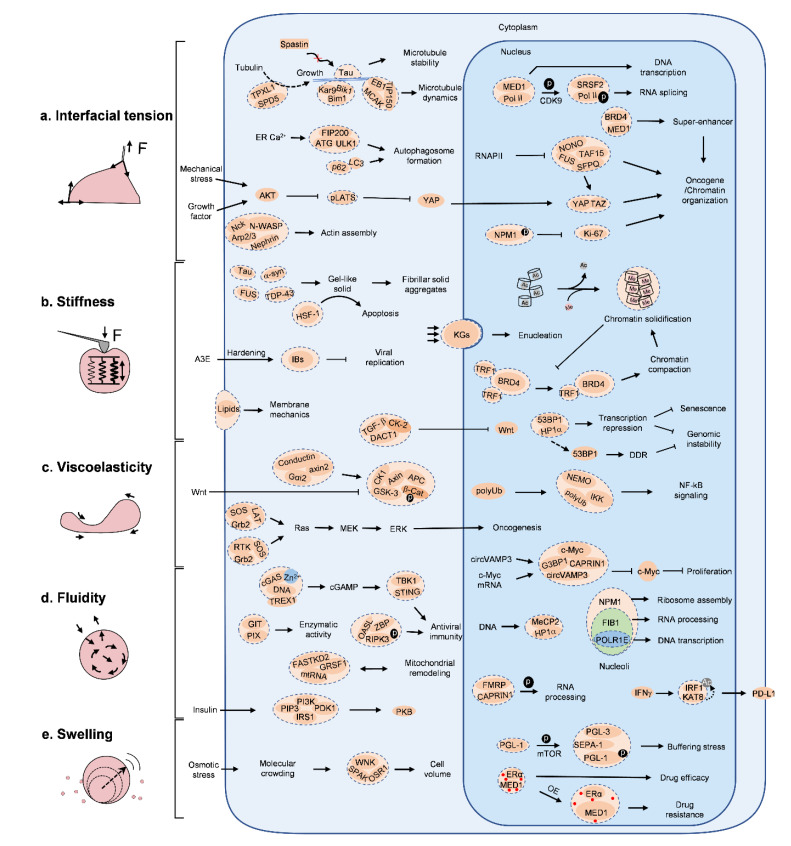
Physical traits of biomolecular condensates on molecular signaling. Biomolecular condensates can modulate molecular signaling pathways through five representative physical traits. (**a**) Interfacial tension. Condensates wet on microtubules, and control their growth, stability and dynamics through the inclusion or exclusion of functional biomolecular partners. Phosphorylation tunes the interfacial tension of Pol II, and thus directs its function to either DNA transcription or RNA splicing. Tension of condensates molds the initiation, elongation, curvature and size of autophagosomal membrane. Interfacial tension of condensates mediates the interactions between transcriptional factors and DNA/RNA, maintains functional genomic assemblies, such as super enhancers, transcriptional hotspots and chromatin organization. Nephrin/Nck1 condensates on membrane enrich N-WASP and Arp2/3 complex and extend their dwell time for actin assembly. (**b**) Stiffness. Condensates of Tau, FUS, α-syn, TDP-43c undergo liquid-to-solid transition, and form abnormal aggregations, resulting in neurodegenerative disorders. Rigid keratohyalin condensates (KGs) are mechanically strong enough to deform the nucleus, leading to enucleation in aquames. Chromatin undergoes a deacetylation and methylation process, and thus behaves as a solid to resist mechanical stress. Lipid clusters on membrane make various domains of stiffness to buffer mechanical perturbations. Targeting physical traits of condensates, for example hardening IB condensates by cyclopamine, has been shown to be feasible to inhibit RSV replication for clinical treatment. (**c**) Viscoelasticity. Viscous TGF-β/DACT1 condensates spatially confine kinase CK-2 in the cytoplasm, thus inhibiting Wnt activation. In the Wnt-β-catenin axis, APC/Axin/CK1/GSK-3 condensates recruit β-Cat for degradation, but when Axin is confined in Wnt/Frizzled/LRP5/6/Dvl condensates, β-Cat enters the nucleus for Wnt gene activation. Ras-ERK signaling requires the continuous retention of LAT or RTK in SOS/Grb2 condensates for downstream activation. (**d**) Fluidity. In the immune response, cGAS senses cytosolic DNA and activates downstream STING signaling by forming condensates, and fluidity can be tuned by Zn^2+^. In nucleoli, it forms three condensed compartments of different fluidity, which show different genomic functions. Similar cases are also seen in versatile condensates. (**e**) Swelling. Condensates buffer the stress-induced molecular crowding by swelling or shrinkage. Swelling of condensates dilutes the concentration of therapeutic drugs, and leads to drug resistance.

First, these small and spherical condensates exhibit a high surface-to-volume ratio so that proper platforms with sufficient interfacial areas are generated for frequent signaling communications [30]. In the case of immune signaling, Nephrin/Nck/N-WASP condensates are formed on the inner side of cell membrane, and host a variety of adaptor proteins and/or effector proteins that can stimulate signaling downstream, such as actin assembly [32] (Figure 3).

Moreover, BCs of this scale also provide a homeostatic internal microenvironment for the protection of central cellular components, and allow for normal biochemical reactions or interactions for molecules with authorized entry, which would otherwise suffer from strong stressful perturbation in chaotic, crowded and dynamic intracellular conditions. Furthermore, to achieve the exquisite control of genomic stability (e.g., RNA protection and DNA repair machinery), cytoplasmic or nuclear condensates are thus extensively involved [33,34,35,36].

Condensates of this scale manifest spontaneous random trajectories in the intracellular scenarios, which is in accordance with the physical concept of Brownian motion [37]. In this way, condensates show increasing possibilities for collision and fusion, thereby contributing to size growth and molecular communications [38]. Notably, because of the heterogeneous contents of BCs and different molecular binding states, constitutive internal particles of condensates also show differential motility and fit with different diffusion models [35]. According to the concept of Brownian motion, particle motility negatively correlates with the size increase. As such, smaller condensates generally show faster motion and higher interfacial tension, while large condensates manifest decreased motility and interfacial tension; as a result, the size of condensates are controlled. Thus, the balance between Brownian motion and interfacial tension depicts one of critical mechanisms for size control of condensates. However, so far, the role of Brownian motion on condensate-dependent cell biology remains mostly underappreciated; it is worth making additional investigations on how Brownian motion interacts with phase separation.

### 3.2. Wetting

Intimate teamwork is established on the basis of molecular proximity. BCs keep close interactions with adjacent cytoskeletal filaments and various membranous organelles, including the endoplasmic reticulum (ER), lysosome, autophagosome, mitochondria and cell membrane, through a physical effect called wetting, or in some cases capillarity.

Wetting, as a physio-chemical concept, describes how a soft liquid droplet contacts a rigid substrate (for example, a glass or plastic surface), or briefly, a liquid–solid interaction. In the case of wetting, the liquid shows a high affinity for the solid substrate, and spreads mostly out on the surface, but with regard to non-wetting, the liquid presents low affinity to the substrate, and instead of spreading, it normally slides on the surface or rests on the solid as a spherical cap, where a parameter termed contact angle, θ, makes a clear definition (θ < 90°, wetting; θ > 90°, non-wetting). The interplay between BCs and adjacent rigid substrates mostly follows this rule, which has been proved in some specific biological scenarios, such as membrane, cytoskeleton and soft matter.

Membrane. Structural and geometric reprogramming of membrane sheets, specifically, the assembly, elongation, bending, fusion, and dynamics, dictate their major characteristics for cell biology. Wetting of BCs plays important roles [22,39,40]. Representatively, the formation of intracellular vesicles (e.g., autophagosomes and synaptic vesicles) illustrates how wetting actively initiates and sculpts membranous sheets [17,41,42]. First, condensates establish a number of interfaces with high surface energy that are prone to adsorb the membrane precursor, for example LC3, for the autophagosome (Figure 2a and Figure 3). Upon membrane enclosure, the balance between the interfacial tension of condensates and membrane strength makes the final determination, that is, when surficial tension is below a certain critical value, it tends to form piecemeal sequestration, partially sequestering droplets, while when above such a value, it forms larger vesicles that fully enclose condensates. In the case of very large condensates, decreased interfacial tension will bend the wetted membrane sheets, and isolate condensates from the bulk by membrane closure, evidenced by the formation of a cup-shaped condensate intermediate [17]. Principally, interfacial tension functionally serves as a mechanical organizer that molds the pre-wetted membrane precursor into double-layered vesicles, and the differential of interfacial tension between condensates and cytosol determines membrane bending orientation towards either droplets or cytosol [41]. Likewise, membrane bending orientation during endocytosis and formation of lamellipodia protrusions for cell migration are well controlled through the wetting of BCs [43].

Cytoskeleton. The cytoskeleton, including actin, the microtubule and intermediate fiber, is an architectural filament and tubule network that extends throughout a cell, underpinning molecular signaling and organelle tethering, as well as a variety of cell behaviors including morphology, adhesion, division and migration. BCs are widely harnessed for cytoskeleton nucleation, growth, rewiring and dynamics [44,45,46,47,48] (Figure 2a and Figure 3). Tau condensates make a typical example [46]. First, Tau condensates recruit tubulin for microtubule bundle nucleation and polymerization; afterwards, they wet mini microtubule bundles and further promote bundle elongation through condensate collision and fusion. Finally, Tau condensates totally wet onto microtubule bundles, and maintain their stability. It is rational to imagine that the complete wetting of Tau condensates on microtubule bundles will endow the surface with a new set of physical features. In some other examples, SPD-5 and Rad52 condensates can, respectively, concentrate tubulin to form microtubule asters in cytoplasm and nucleus, during which wetting ensures the robust interactions between the condensates and microtubule [49,50]; EB1 condensates guide microtubule plus-end dynamics [51]; and nephrin/Nck1 clusters at the cell periphery organize actin assembly, and drive the formation of dense filopodia [52].

Soft matter. Apart from rigid membranes and cytoskeletal filaments, wetting also influences the interplay between condensates and soft matter in cells, including heterotopic condensates and genomic loci. Due to the differential surface physics of multiple heterotopic condensates, intracellular condensates manifest polymorphism, as indicated by the formation of monophasic condensates, core/shell or core/shell/shell condensates, and hollow condensates [15,31,53] (Figure 2a and Figure 3). Typically, in the nucleolus, NPM1, FIB1 and POLR1E construct multi-layered condensates, and modulate three distinct functions (i.e., ribosome assemble, rRNA processing and rDNA transcription) in each compartment, collectively elucidating the delicate spatiotemporal control of ribosome biogenesis [31], which is reminiscent of ATP-modulated core/shell stress granules and transcriptional condensates [54,55]. Of note, upon collision, differences in wetting lead heterotopic condensates to have complete fusion, partial fusion, or no fusion, which further affects the efficiency of substantial exchange and relevant compartmentalized functions [31,56]. Mechanistically, condensates wetted onto chromatin can work as knobs; upon collision and fusion, forces exert on such knobs and drag targeted chromatin together, restructuring the genomic landscape [19].

## 4. Stiffness

Stiffness, or in the other extreme, softness, defines the mechanical strength of a material. Building up through an interactive network of molecular components, BCs display a broad range of stiffnesses, and affect subsequent biological functions (Figure 2b and Figure 3).

### 4.1. Mechanical Strength

It is rational to accept that stiff condensates are able to resist mechanical strains and maintain their constitutive and structural integrity, and upon direct contact they are further capable of restructuring the adjacent environment, while the softs are prone to being passively deformed or compressed [4,49,53,57].

The nucleus houses the genomic information of a cell and modulates cell commitment. But to acquire a more specific cell identity, for example, red blood cells and squames in the skin, cells need to remove the nucleus, which is termed enucleation [2]. As reported, during the differentiation of epidermal keratinocytes towards the epidermis, filaggrin initially partitions into keratohyalin granules (KGs), and crowds the cytoplasm with a burst of rigid KGs. In the next step, KGs deform the nucleus through robust mechanical compaction, where KGs assembled by wildtype filaggrin display pronounced stiffness (with an average Young’s modulus of around 6 kPa) to deform the nucleus, but those of tail-deficient mutants are several times softer (around 1.5 kPa in average) and fail to deform the nucleus.

In the nucleus, chromatins form rigid solid-like condensates, which have a greater ability to resist mechanical stress than those in the dispersed state [4]. Likewise, some other densely compacted condensates, such as AKAP95, Xist, and Swi6, further demonstrate the critical role of stiffness in controlling transcriptional activity, spatial distribution and chromatin organization [58,59,60].

More broadly, the phase separation of lipids (e.g., phosphocholine, sphingomyelin, and cholesterol) on the cell membrane creates micrometer-size domains of various stiffness that constitute the overall physical features of the membrane, dictating signal transduction, membrane trafficking and immune responses [61,62,63].

### 4.2. Steric Blockage

Rigid condensates, for example, nuclear speckles, can function as insulators that spatially segregate the genome into separated topological domains for diverse functions [64,65]. Particularly, with the loss of the nuclear envelope in cell division, vulnerable nuclear components are exposed to cytosolic hazardous risks; in this regard, for example, LEM2 condensates have been reported to mediate nuclear envelope reformation and function as a temporary shield for chromatin organization [6]. In addition, in some other pathological conditions, for example, neurodegenerative disorders, α-synuclein, FUS, and synthetic condensates undergo a soft-to-stiff transition and gradually form rigid amyloid hydrogel or fibrous aggregations introducing cytotoxicity [66,67,68]. Indeed, stiff and inert condensates that are hard to be degraded overconsume spatial resources and block normal signaling axis in cells, thus steering cells towards dysfunction.

### 4.3. Molecular Activity

In response to some unfavorable external triggers, such as heat, pH, energy and osmotic stresses, cells can either shift into a quiescent or dormant state, with reduced metabolic activities through the formation of stiff condensates [69], or enhance their own activities to actively buffer or escape the harsh environment by generation of a set of soft and active condensates [28]. Inspired by the inert property of stiff condensates, hardening the soft and disease-driven condensates, either by mutation in key residues or using hardening reagents (e.g., steroidal alkaloid cyclopamine and its analogues) proves to be a promising strategy aiming at undruggable targets for medicine design and development in clinics [20,59].

## 5. Viscoelasticity

Viscoelasticity is a commonly used physical parameter to describe the rheological characteristics of a material under mechanical stress; normally, viscosity depicts a long-term effect for a liquid, while elasticity depicts that for a solid at short timescales. The real-world panorama of a live cell is a bustling, dynamic, and stressful scene, with physical fluctuations (e.g., traction, friction, and shear) from substance transport and cytoskeleton dynamics, as well as a variety of biochemical disturbances (e.g., ROS). Therefore, it needs a proper force to maintain a stable microenvironment for the smooth operation of a cell event, and the viscoelasticity of BCs makes a great contribution [70,71]. Viscosity ensures condensates pack tightly interacted constitutive components together, and elasticity guarantees that condensates relax back to their original morphology after the withdrawal of external forces [1,72] (Figure 2c).

As reported, the viscosity of BCs is in the range of 0.7–30 Pa·s, comparable to colloid liquids, but a thousand times larger than that of cytosol [1,24,31,72,73]. These viscous condensates can function as stable intracellular hotspots that harbor a variety of specific molecules for functions [72] (Figure 3). For example, through the formation of LAT-Grb2-SOS condensates on the inner leaflet of the cell membrane, the dwell time of SOS on the membrane is markedly elongated, which promotes downstream Ras activation [74]. A similar phenomenon also occurs in N-WASP and Arp2/3 condensates on the membrane for actin assembly [32]. The dwell time, in some cases called the residence time, rules the feature of binding site occupancy, and affects the overall biological functions [75]. In particular, in neuron cells, transporting a number of essential proteins to distal regions, especially across the long and narrow axon, is challenging. Packing the mRNAs of required proteins into viscoelastic RNA granules and hitchhiking moving lysosomes through the tethering of other ANXA11 condensates for transport can be a smart and feasible choice. In this case, once mRNA transcripts arrive at destinations, they are unloaded and then transcribed into coded proteins. During long-distance transport, viscoelasticity buffers tensile forces from the lysosome and shear from the cytosol [76]. In some more direct in vitro examples, viscoelasticity from protein–DNA co-condensates generate a force of 0.2–0.6 nN, which has proven to be sufficient to overcome the entropic tension of the non-interacted DNA (at the pN level), and drag them into close vicinity for pairing [77], which is in line with the functional observations of transcriptional condensates [19,38,78,79,80].

From a view of material physics, protein condensates have been shown to be viscoelastic Maxwell fluids, whose viscosity strongly increases as a function of time (termed aging) [81]. This may offer an explanation on how phase separation contributes to DNA compaction, such as HP1α droplet-induced DNA compaction in heterochromatin formation [82].

## 6. Fluidity

As active and dynamic assemblies, BCs represent a liquid-like property, which can be defined as fluidity. In a fluid, van der Waals forces dominantly maintain molecular interactions, so that molecules do not interact as robustly as in a solid. They are only orderly organized in some restricted spatiotemporal regions, whose boundary and size change constantly along with restless collapse and reconstruction. Highly fluidic condensates exhibit frequent molecular motion, and the interactions can be vividly depicted as a “kiss and run” mode, while those of low fluidity are commonly stationary and inactive condensates. Furthermore, fluidic condensates are prone to be deformed or shed mini droplets in response to mechanical stress, and fuse with each other upon collision; but those of low fluidity are reluctant to move or be deformed. Collectively, fluidity constitutes one of biological landscapes of condensates for functions, whose aberration, either activated or inactivated by mutation or alteration in composition, can lead to pathological disorders (Figure 2d and Figure 3).

For instance, in response to DNA or Zn^2+^, cGAS undergoes phase separation and forms liquid-like condensates, leading to enhanced enzymatic activity for innate immune signaling [83]. In normal physiological conditions, TREX1 forms relatively lower fluidic external condensate shell surrounding the cGAS/DNA core; thus, due to the reduced access to DNA interior, its activity is remarkably mitigated, while in pathologic conditions (e.g., Aicardi–Goutières syndrome), the TREX1 mutation E198K leads to the formation of more dynamic TREX1/cGAS/DNA co-condensates driving internal DNA degradation, and ultimately suppresses STING signaling [84]. Consistently, some other negative regulators of the cGAS–STING axis, such as barrier-to-autointegration factor 1 (BAF), exhibit same physical traits. Overall, this switch-like model between one-phase spherical condensates and multi-layer core/shell-structured condensates represents how fluidity and viscoelasticity participate in the control of the spatial localization of DNA, cGAS and related negative regulators, and this paradigm illustrates a general mechanism that commonly applies to some other immune-sensing pathways, like inflammasome signaling [85]. In addition, in the nucleus, transcription factors (TFs) can form transient condensates at native genomic loci, and serve as “active hubs” with highly dynamic, sequence-specific interactions recruiting RNA polymerase II (RNA Pol II), and activating transcription [12,79,86]. In the control of chromatin, fluidity determines the chromatin interaction ability for proteostasis [87]. By contrast, as BCs mature and form a gel-like or solid-like organization, their drastically reduced fluidity disturbs normal functions.

### Swelling

In BCs, constitutive molecules are frequently recruited into and released out of condensates, so that volumes of condensates fluctuate greatly, thereby leading to swelling or shrinkage in volume. In the context of hyperosmotic stress, the cytoplasm is subjected to molecular crowding and osmolarity changes, a set of condensates (e.g., WNK, ASK3, DCP1A, and YAP condensates) form immediately and buffer intracellular osmolality and/or balance protein stress, as a contingency mechanism for cells, until the initiation of protective measures [10,27,28,88] (Figure 2e).

From another point of view, with swelling or shrinkage, molecular concentration and subsequent activity in condensates are considerably re-orchestrated. As reported, an increase in volume and the recruitment of inert clients synergistically reduce molecular concentration and introduce steric hinderance for function [7], whereas the exclusion of non-functional clients in shrinking condensates significantly enhances activity and specificity, as a result leading to functional compartments in cells. Altogether, the swelling and shrinkage features of condensates maintain the homeostasis of intracellular environments under stress.

## 7. The Interplay of Physical Traits

As discussed, interfacial tension, stiffness, viscoelasticity, fluidity and swelling represent major physical traits of BCs. Although introduced separately, however, in the dynamic and constantly changing biological systems, they inevitably co-exist and are intensely interdependent (Figure 2), which representatively shows in the following scenarios: (1) to mechanically deform an adjacent structure, it needs condensates with proper stiffness, but it firstly requires condensates to wet on the material surface, and grow into a proper volume either by fusion or swelling; (2) to buffer crowding molecular stress, condensates swell or shrink, but their fluidity allows for appropriate molecular throughput, thereby in return reshaping the stiffness and viscoelasticity; (3) genome restructuring needs interfacial tension and viscoelasticity to spatiotemporally regulate targeted chromatins, but also fine-tuned stiffness and fluidity are harnessed to combat against mechanical compaction and benefit transcription in the nucleus; (4) under shear from the flow of the cytosol, viscoelasticity and fluidity allow for an active and stable environment inside condensates; and (5) high stiffness and reduced fluidity lead to pathological disorders or cell dormancy.

Although numerous studies have highlighted the physical traits of BCs, the concepts are usually poorly defined, and sometimes also misunderstood. As it should be, it is impossible that only one physical trait comes into effect, but in future studies, a clear and accurate definition of the physical traits of condensates and figuring out their relationship are urgently needed; in particular, it will require a more comprehensive understanding on the association between physical traits and biological functions, as well as the molecular basis.

## 8. Determinants for Condensate Physics

**Figure 4:**
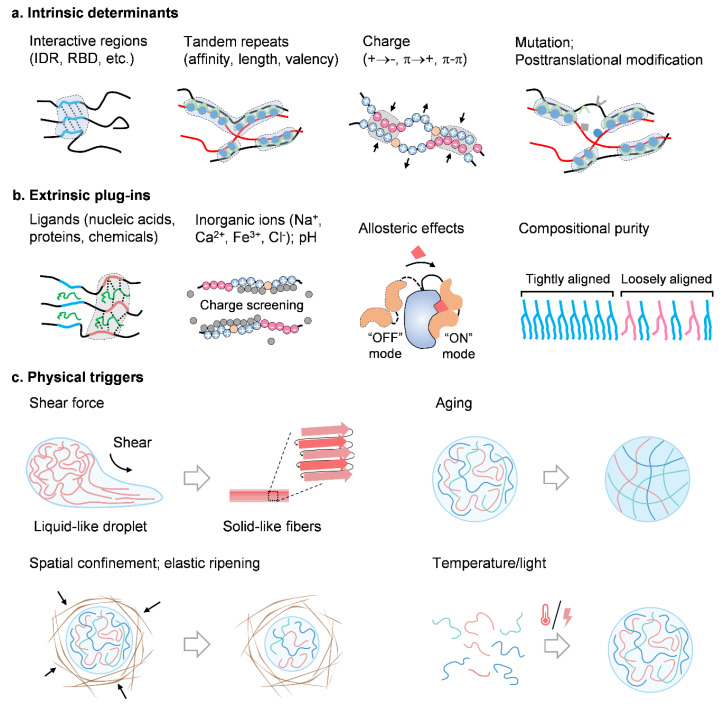
Determinants for physical traits of biomolecular condensates. Based on recent reports, determinants that modulate physical traits of biomolecular condensates can be mainly summarized into three categories, that is, intrinsic determinants, extrinsic molecular plug-ins, and some physical triggers. (**a**) Intrinsic determinants refer to the molecular characteristics that drive the formation of condensates, of which interactive regions, tandem repeats, charge, and some mutations as well as posttranslational modifications exert major contributions. Condensates generate on the basis of weak multi-valent interactions; in particular, IDR, RBD and PLD are the most typical interactive regions of condensed biomolecules, as these regions commonly allow for the multi-valent molecular interactions. Moreover, the affinity, length, and valency of these interactive regions rule the overall traits of the condensates. (**b**) Biomolecular condensates are frequently affected by some extrinsic factors in the crowded and complex intracellular environment, for example, ligands, metal ions, pH, chemical reagents and fluctuations in composition purity. These factors enhance or compromise the intrinsic features of biomolecules, and thus give rise to the final properties of condensates. (**c**) Except chemical and biological factors, some physical triggers can also remodel physical features of condensates. Directional shear fosters ordered alignment of compositional architecture that shifts liquid-like droplets into solid-like fibers. Aging, a structural rearrangement over time, leads to a liquid-to-solid transition of condensates, and results in gel-like or glass-like traits of condensates. Spatial confinement, for example being surrounded by cytoskeletal fibers, disturbs the molecular interaction, and through a process called elastic ripening, size and composition of condensates display another scenery compared with those in free conditions. Some other factors like temperature and light will also tune condensate physics by virtue of altered molecular interactions.

The physical traits of condensates are generally established on the mesoscopic level, but the determinants lie at the molecular level, where multivalent weak secondary forces, namely, van der Waals forces, hydrogen bonds, hydrophobic forces, salt bridges, cation–π interactions, and aromatic ring stacking, matter. In this section, we summarize the latest knowledge on the main drivers of condensate physics (Figure 4). Together, these factors can be divided into three categories: intrinsic determinants (i.e., sequence, length, charge, conformation, mutations, posttranslational modifications (PTMs), and affinity), extrinsic plug-ins (i.e., nucleic acids, proteins, inorganic ions, pH, and some small chemicals [20,89]), and physical triggers (i.e., shear, aging, spatial confinement, temperature [90], and light [19]).

### 8.1. Intrinsic Determinants

Molecular structures and intermolecular interactions make the roots of the overall physical traits of BCs (Figure 4a). Foremost, the intrinsic molecular characteristics, such as intrinsically disordered regions (IDRs), were originally considered as the main driver for phase separation [10,91,92]. With such domains, proteins fail to form highly folded tertiary structures, but some weak secondary structures (e.g., random coils and a β-sheet), thus defining the molecular grammar for interfacial tension, stiffness, viscoelasticity, fluidity as well as swelling of condensates. As research advances, such condensation-driving regions extend to some other highly interacted domains, such as RNA-binding domains (RBD), prion-like domains (PLD), zinc finger domains (ZFD), Tudor domains, pseudo-repeat regions [93], etc. These regions or domains normally harbor protein motifs of several tens of amino acids, that can introduce individualized preferences for the intermolecular interactions.

In the case of FUS family proteins, glycine, glutamine and serine in both RBDs and PLDs are the main controllers for phase separation, and particularly, glycine modulates fluidity, whereas glutamine and serine modulate stiffness [94]. In the case of polypeptide condensates, those of arginine have a viscosity approximately 100 fold greater than those of poly-lysine [95]. Moreover, tyrosine, phenylalanine, and arginine drive elasticity of polypeptide–RNA condensates, while lysine, proline, and serine residues determine viscosity [96]. It should be noted that different condensates are made of different components; therefore, the molecular profiles vary significantly, so that all of above-mentioned conclusions should be considered based on the molecular contexts.

In some other concepts, such as scaffold–client interaction, sticker–spacer identification, and cation–π and π-π attraction, the length of the interactive unit as well as its distribution across the sequence determine multi-valent binding patterns for phase separation [97,98,99,100,101]. Mutations and PTMs that alter the interacting activity will reorchestrate condensate features, resulting in changes in function. As in the example of RNA polymerase II (Pol II), phosphorylation of the C-terminal domain regulates mediator condensates to be transcriptional or splicing [102]. Interaction of tumor suppressor SPOP and DAXX in nuclear condensates contributes to enhanced enzymatic activity, but mutations in SPOP disrupt phase separation and correlate to loss-of-function and oncogenesis [11].

### 8.2. Extrinsic Plug-Ins

Apart from the intrinsic biomolecular profiles, a variety of extrinsic elements are able to plug in the condensates and rule the physical traits. These biological plug-ins involve nucleic acids (e.g., short vs. long, single vs. double stranded, structured vs. unstructured, DNA vs. RNA) [103], proteins, therapeutic chemicals, and a variety of inorganic ions. Exposed to these extrinsic plug-ins, molecular panorama of condensates is tremendously remodeled, thus giving rise to another physical scenery (Figure 4b).

For example, long noncoding RNA (lncRNA) *SLERT* has been proven to modulate the molecule compactness of DEAD-box RNA helicase DDX21 by softening the rigid DDX21 condensates, facilitating Pol I processivity and rDNA transcription. By contrast, *SLERT* loss or structural mutation leads to stiff DDX21 condensates with inactivated activity for ribosomal RNA production [104]. Indeed, RNA has been widely reported to prevent condensate solidification [12,72,79,105,106]. In the cGAS–STING axis, long DNAs more efficiently tune the activity of cGAS condensates than short ones [83], due to longer tandem binding units and more robust DNA-cGAS multivalent interactions [26,107]. With regard to the structures and types of nucleic acids, unstructured DNAs or RNAs (or linear nucleic acids) show almost identical effects, but the stem-loop structured DNA/RNA leads to more viscous condensates than that of unstructured DNA/RNA [96]. In some other contexts, for example, the insertion of RNA controls the condensate size [108] and also compromises the viscosity of the condensates [109]. The utilization of therapeutic chemicals switches active soft condensates into inert rigid ones for clinical benefits [20]. Exploring the stoichiometry between proteins and ligands would be an important and interesting issue on how ligand insertion-remodeled physics affects cell biology.

Charge is one of crucial factors that modulate molecular interactions, and cells have evolved a variety of charged domains and some metal binding sites in proteins to meet the molecule bonding requirements. Intracellular fluxes of metal ions (such as, sodium, potassium, calcium, and zinc) would either enhance or block the charge of proteins, and finally change the physics of condensates [109,110]. As mentioned above, once the proper amount of Zn^2+^ is recruited, cGAS condensates are stabilized and cGAS enzymatic activity is enhanced. In Tau and CTTNBP2 condensates, it is Zn^2+^, but not Mn^2+^, Fe^2+^, Co^2+^, Ni^2+^, and Cu^2+^, that shifts the propensity of phase separation on the basis of zinc-induced high-order assemblies [93,111]. Ca^2+^ induces condensates on the surface of ER [112]. Moreover, ions can also facilitate a liquid-to-solid transition that induces the generation of aberrant aggregations [113]. To date, how metal ions interact with proteins, in terms of binding sites, stoichiometry, and affinity, as well as the ion dynamics in condensates, are still elusive. Elucidating the multiple roles of ions in condensate physics, as well as their association with biological significance, will provide more insights into physiological and pathological conditions.

External plug-ins can come into effect by binding onto the inactive site of proteins, but still induce direct protein–ligand recognition, which can be described as allosteric effects. As indicated, SHP2 mutants recruit widetype SHP2 to form condensates, and activate the ERK-MAPK axis. However, once the allosteric inhibitor SHP099 binds onto protein tyrosine phosphatase (PTP), condensates of SHP2 mutants are strongly diminished, which enhances SHP2-PTP activity [89].

Molecule insertion can also change the compositional purity of condensates, whose dynamic fluctuation can serve as a functional switch for condensate physics. For example, lipids of different structures can be sorted into different sub-locations through phase separation. The insertion of short and branched lipids is able to shift the tightly compacted condensates of linear lipids into loosely organized soft ones, and thus result in decreased local stiffness that disrupts membrane integrity [114,115]. In general, the more complex the composition, the more active the internal molecular activities, but with a reduction in overall stiffness, which can be explained by compromise of strong protein–protein interactions by molecular insertion.

### 8.3. Physical Triggers

The overall physical traits of condensates are derived from the microscopic characteristics of molecular assemblies. Except for chemical and biological drivers, some external physical triggers, like shear force, aging, spatial confinement and fluctuations of temperature and light, can also serve as alternative but undeniable inducing elements of BCs (Figure 4c).

### 8.4. Shear Force

An external applied shear allows biomolecules to move bidirectionally and results in more ordered molecular alignments. As reported, when a shear force, respectively, applies to FUS, Ded1, Annexin A11, zFF, and silk condensates, all such condensates undergo microarchitectural re-organization and thus shift liquid-like condensates into solids with elevated stiffness. By assembling into a β-sheet network, the mechanical strength of solid FUS condensates is even comparable to that of dry silk fiber [3]. In some intracellular occasions, such as the stirring of the cytoskeleton and matter transport in the cytoplasm, such induced shears are able to govern the fission and fusion of BCs; thus, interfacial tension and fluidity are passively re-organized [23].

### 8.5. Aging

BCs can change their physical traits with time, which is reminiscent of the typical physical concept termed aging. As has been widely reported, condensates can undergo a liquid–solid transition through either gelation or glass-like aging [81]. Notably, these two concepts should be carefully distinguished, as gelation undergoes a sudden transition from a viscoelastic fluid to a solid when it reaches a critical point, but glass-like aging shows no such sudden change. At all aging stages, condensates behave as viscoelastic Maxwell fluids, where viscosity increases evidently while elasticity fluctuates weakly. In addition, the mechanical strength of condensates increases strongly, as in the cases of α-synuclein, FG, and SPD-5 condensates, particularly when they shift into fibers or a crystal-like state [49,66,116,117].

### 8.6. Spatial Confinement

The cytoplasm is a complex and crowded environment. In the study of BCs, one also needs to consider the adjacent biological surroundings. For example, keratins, as the backbones of intermediate filaments, can form biological cages around filaggrin condensates that shift the active and highly mobile condensates into inert and stationary ones, so that it impedes the intrinsic interfacial tension and stiffness of condensates for function [2]. Furthermore, being isolated from the source of biomolecular components by spatial confinement, condensates not only fail to function properly, but also shrink or totally return into the mixing phase, which can be explained by the concept of Ostwald ripening and/or elastic ripening [38].

### 8.7. Others

Some other physical triggers, like temperature and light, can greatly coordinate molecular behavior and determine condensate physics. However, as it has been widely discussed elsewhere, we do not further discuss this here.

Considering the heterogeneous composition and features of condensates and the still-limited number of study reports, it is arbitrary to make conclusions on which single element contributes to each physical trait of condensates. Future studies will need to perform more comprehensive research on determinants at the molecular level, and decipher how condensates function or dysfunction in organisms.

## 9. Clinical Significance of Condensate Physics

Genetic variations and molecular signaling dysregulation are generally considered the main reasons for the onset and progression of diseases. The latest and future therapeutic strategies for personalized treatment and precision medicine will heavily rely on the comprehensive awareness of molecular profiles, signaling networks, and working modes. Hitherto, it has been reported that a broad variety of biomolecules can form condensates, and these molecular profiles interestingly overlap well with those of great clinical significance [118,119,120], as summarized in Figure 1 and Table 1. Thus, condensate physics-guided molecular behavior represents a novel but robust insight to decipher the molecular codes in diseases and medical treatment.

In terms of pathogenesis, for example, hereditary synpolydactyly, and disease-associated amino acid repeat expansions have been found in the IDRs of multiple transcriptional factors, which drive the unblending of the transcriptional co-condensates due to the changed interfacial tension, and thus initiate disease-leading transcriptional programs [55]. More broadly, a subset of proteins (HMGB1, RUNX1, CALR, etc.) with a mutant tail in IDRs drive the spatiotemporal re-organization of nucleolus granules in a similar way, and lead to a variety of rare genetic diseases (including brachyphalangy, polydactyly and tibial aplasia syndrome) [125]. Moreover, stiff α-synuclein, FUS, and Tau condensates, as well as their induced fibrous solid aggregations, lead to strong cytotoxicity in neurodegenerative disorders; the stiffness-remodeled condensates can also regulate molecular activity and genomic accessibility in inflammasome activation and cancer progression [117]. In addition, the fluidity- and viscoelasticity-mediated elongation of molecule dwelling on the cell membrane help to activate the downstream Ras/AMPK axis for tumorigenesis. Together, these lines of evidence indicate the driving roles of condensate physics in disease onset and progression. In response to the volume change by droplet fusion/fission or swelling/shrinkage, condensates can also dilute or concentrate the involved drugs, which affects drug resistance or efficacy [7]. In return, by adjusting the doses of small chemicals, the mixing or demixing fate of condensates can be modulated [129].

**Figure 5:**
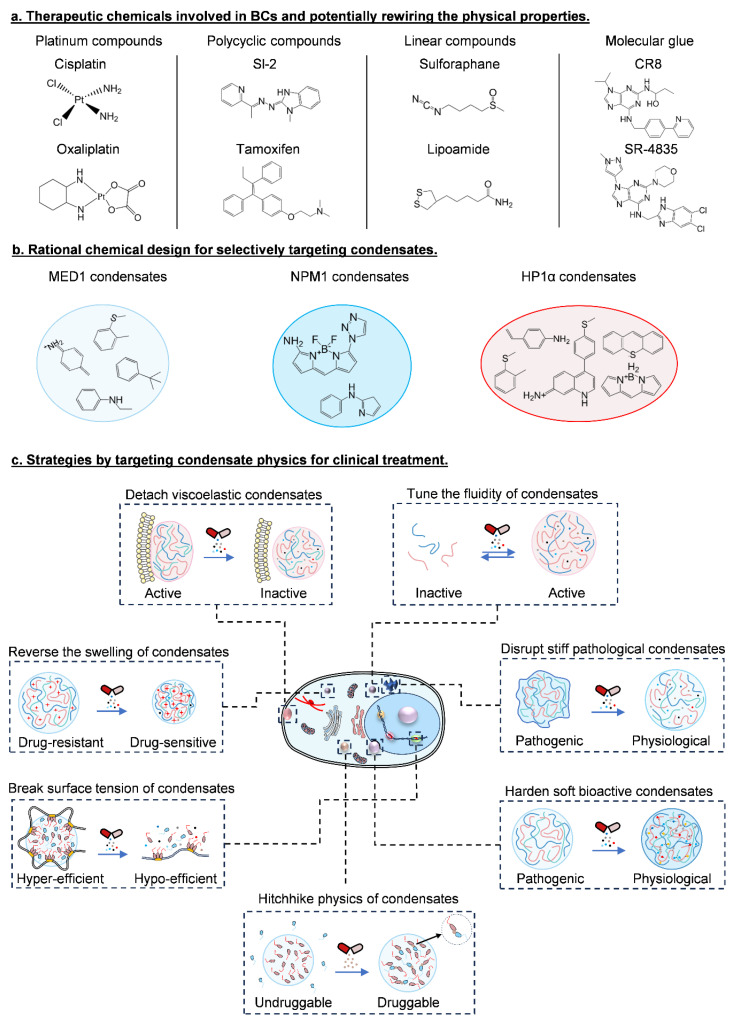
Therapeutic chemicals and their selective interaction with BCs, and next-generation treatment by targeting condensate physics for clinical benefit. (**a**) Representative types of chemicals that are involved in BCs and thereby rewire the physical traits of BCs, namely, platinum-chelated compounds, polycyclic compounds, linear compounds, and molecular glue. (**b**) Examples of BCs that are selective to functional groups in therapeutic chemicals. (**c**) Future strategies that target condensate physics for clinical benefits.

With regard to medical treatment, it always needs to efficiently and precisely deliver the proper number of drugs to specific spatiotemporal location with robust and durable action. In this regard, several critical parameters, such as target druggability, drug stability and solubility, effective concentrations, biocompatibility, and pharmacokinetics should be considered to evaluate the overall therapeutic efficacy and safety. By compartmentalizing therapeutic agents into specific condensates, it significantly reshapes drug efficacy and may help evolve next-generation therapeutics (Figure 5). Indeed, recruiting a variety of widely used small-molecule therapeutics (e.g., cisplatin, THZ1, JQ1 and mitoxantrone) into nuclear condensates of BRD4, HP1⍺ and NPM1 [7] drastically alters the stiffness and fluidity of condensates, leading to a sharp attenuation of protein activity [20]. As reported, MED1 condensates prefer aromatic rings, cationic amines and their N-acetyl propylamine derivatives, while NPM1 prefer aromatic and amine rich moieties and HP1α prefer BODIPY and xanthene dyes [130] (Figure 5a,b). Therefore, therapeutics can be designed to achieve clinical benefits by specifically targeting condensate physics, either by promoting condensate formation or dissolving pathological condensates [16,131,132,133] (Figure 5c).

In the emerging field of molecular glue, the degradation of tumorigenic proteins, like CCNK, CDK12/13, and cyclin K, shows great survival benefits [134,135,136]. Intriguingly, such proteins also exhibit a wide range of IDRs, which may imply the potential contribution of phase separation. Indeed, the viscoelasticity and fluidity of BCs show similar characteristics as a “glue”, which can be used as a promising strategy for the design of high-performance degraders in medicine. Thus, targeting phase-separated condensates proves a feasible strategy to achieve treatment benefits [137].

## 10. Conclusions and Perspectives

**Figure 6:**
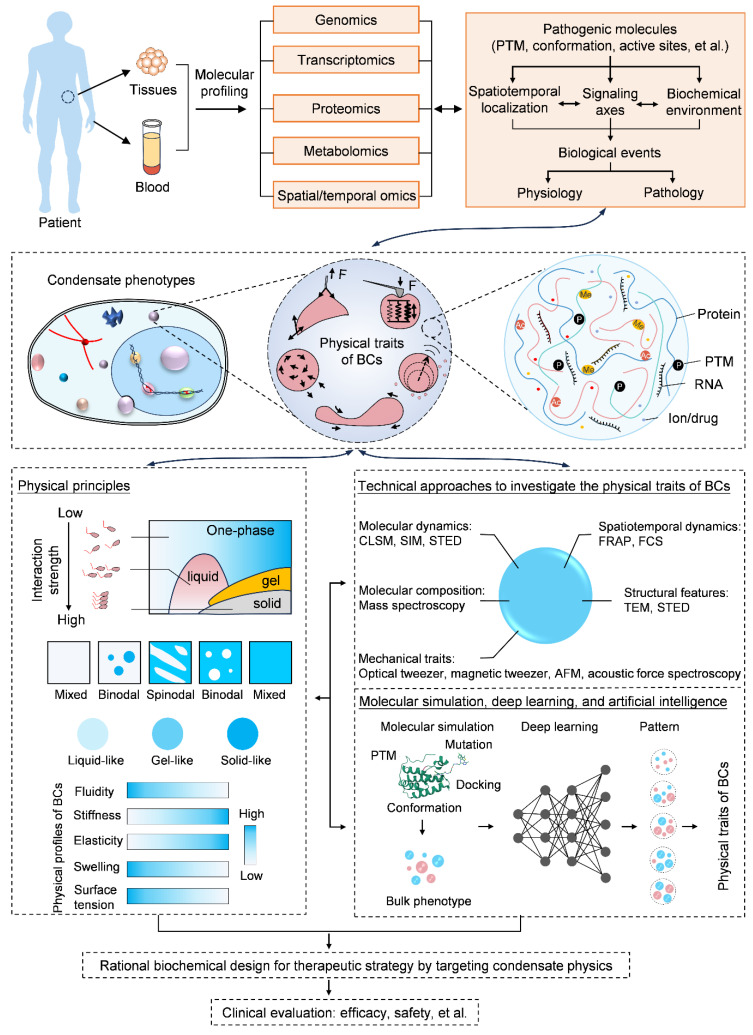
Future challenges and opportunities by targeting condensate physics for therapeutic purposes. Diseases commonly originate from dysregulated biomolecular signaling. Techniques of omics together with traditional molecular biology elucidate pathological fundamentals. From a new perspective, the physical traits of BCs become one of the main drivers. To meet the tremendous requirements and bypass the long-lasting bottlenecks in clinics, the rational design of therapeutics by targeting condensate physics offers new opportunities and relies on some basic awareness of BCs. First, some physical models on the molecular interaction strength, coalescent states and physical features of BCs should be established corresponding to each biomolecular identity, especially regarding the commonly used therapeutic chemicals. Second, more advanced techniques should be designed and incorporated to investigate the interior molecular behaviors of BCs, in terms of the molecular content, dynamics, microscopic forces etc. Third, considering the complexity and transient motion of diverse internal biomolecules, by using molecular simulation, deep learning and artificial intelligence, they offered an in-detailed basis for BCs. In particular, combining physical theory and these state-of-the-art techniques, the contribution of condensate physics to biomolecular activity and significance in pathological development and prognosis will be clarified, which, in return, will benefit disease treatment.

Reconsidering the biological effects of BCs through a physical perspective refreshes our current knowledge of molecular behaviors, and it also offers us deeper insights into how biomolecules dictate cell fate and lead to diseases. To date, despite an intensive body of studies involved in this research hotspot, there are still a myriad of unknowns, both challenges and opportunities, ahead. Seeking a solution for clinical problems will rely on multi-disciplinary cooperation, where the techniques of omics and molecular cell biology uncover potential molecular profiles and signaling in the pathogenesis of patients, while physical principles and state-of-the-art instruments further demonstrate the contribution of molecular behaviors and condensate physics for diseases (Figure 6). Indeed, it needs a more sound and accurate definition for the physical traits of condensates in the intracellular milieu; armed with the state-of-the-art techniques to sketch out the physical parameters of condensates in situ and thereby elucidating the corresponding biological effects would be another crucial issue of concern. Importantly, given the complexity and dynamic variation in components of condensates at different times and spaces, clarifying the whole constitutes and delineating how such ever-changing elements work together to make the overall physics of condensates and participate in the regulation of biological functions will be a great field in which to decipher the molecular codes. Indeed, as a frontier field embracing multi-disciplines, by making the most of the techniques of omics, machine learning, and big data analysis, it will offer alternative opportunities to obtain a comprehensive scope of the condensate-associated biophysics. Finally, linking the well-understood physical traits of condensates to the rational design of medicine and exploring the translational potentials may be more conducive to the development of precise medicine and improve clinical outcomes.

## Figures and Tables

**Table 1:** Clinical significance of physical traits of biomolecular condensates.

Protein	Diseases	Location	Physical Traits	Description	Refs
IRS1	Diabetes	Cytosol	Fluidity	IRS1 condensates mediate insulin signaling via recruiting PI3K, PDK1, PIP3 and PKB, whose formation is impaired in insulin resistant cells.	[121]
MeCP2	Rett syndrome	Nucleus	Fluidity	MeCP2 condensates selectively concentrate heterochromatin cofactors rather than components of euchromatin. Mutations in MECP2 disrupt MeCP2 condensates, leading to Rett syndrome.	[122]
MLL4	Kabuki syndrome	Nucleus	Interfacial tension	MLL4 condensates maintain the balance between transcriptional and PcG condensates; MLL4 LoF increased chromatin compaction and disrupted nuclear mechanics and architecture.	[123]
MYO7A, USH1C, ANKS4B, USH1G	Usher syndrome	Cytosol	Wetting	Densely packed MYO7A/USH1C/USH1G condensates stabilize tip-links in intestine microvilli and stereocilia. MYO7A mutations disrupt the binding of the motor to USH1 and impair condensates formation.	[124]
HOXD13, HMGB1	Synpolydactyly	Nucleus	Interfacial tension	Alanine repeat expansions enhance the phase separation capacity of the HOXD13 IDR, and the IDR mutant unblend HOXD13 from transcriptional co-condensates, leading to disease phenotype.	[55,125]
SUMO-SIM	AD	Cytosol	Stiffness	Mechanical compression from molecular crowding shapes stiffness of condensates, therefore leading to phosphoregulatory network rewiring.	[67]
FUS	ALS	Nucleus	Fluidity, stiffness	FUS normally operate as liquid droplets, solid aggregation leads to ALS.	[68]
ApoE2, p62	AMD	Cytosol	Universal	Mitochondrial injury drives phase separation of ApoE2 and p62 that nucleate drusen and regulate autophagy, respectively.	[126]
IB	RSV	Nucleus	Stiffness	A3E and cyclopamine inhibit RSV replication by hardening IB condensates.	[20]
VGLL3	Cardiac fibrosis	Nucleus	Viscoelasticity	VGLL3 is incorporated into non-paraspeckle NONO condensates containing EWSR1 and suppresses miR-29b.	[127]
WNK	Stroke, hypertension, hyperkalemia	Cytosol	Swelling	WNK kinases sense molecular crowding and rescue cell volume via phase separation	[28]
MED1-BRD4, HP1⍺, SRSF2, FIB1, NPM1	Cancer	Nucleus	Fluidity, swelling	Drug partitioning into nuclear condensates influences drug concentration and activity: swelling of MED1 condensates induces tamoxifen resistance; ER mutation alters drug affinity.	[7]
p53	Cancer	Nucleus	Interfacial tension	p53 mediates the interplay of nuclear speckles and p21 for gene expression through DNA binding. p53 mutant R248Q condensates host and facilitate the nucleation of amyloid fibrils in cancer cells.	[128]
BRD4-MED1, TAF15, EWS, Sp1	Cancer	Nucleus	Fluidity, wetting	Nuclear condensates create a dense phase with high concentration of transcriptional machinery, serving as interaction hubs for robust gene expression.	[12,79,86]
